# Effect of hydrogen on magnetic properties in MgO studied by first-principles calculations and experiments

**DOI:** 10.1038/s41598-022-13949-w

**Published:** 2022-06-16

**Authors:** Ittipon Fongkaew, Benjaporn Yotburut, Wutthigrai Sailuam, Warakorn Jindata, Theerawee Thiwatwaranikul, Atchara Khamkongkaeo, Nattapong Chuewangkam, Nantawat Tanapongpisit, Wittawat Saenrang, Rapee Utke, Prasit Thongbai, Supree Pinitsoontorn, Sukit Limpijumnong, Worawat Meevasana

**Affiliations:** 1 School of Physics, Suranaree University of Technology, 111 University Ave., Nakhon Ratchasima, 30000 Thailand; 2 Department of Applied Physics, Faculty of Engineering, Rajamangala University of Technology ISAN (Khon Kaen Campus), Khon Kaen, 40000 Thailand; 3 Department of Metallurgical Engineering, Faculty of Engineering, Chulalongkorn University, Phayathai Road, Wangmai, Pathumwan, Bangkok, 10330 Thailand; 4 Department of Physics, Faculty of Science, Khon Kaen University, Khon Kaen, 40002 Thailand; 5 School of Chemistry, Suranaree University of Technology, Nakhon Ratchasima, 30000 Thailand; 6 Thailand Center of Excellence in Physics (ThEP), MHESI, Bangkok, 10400 Thailand

**Keywords:** Ferromagnetism, Magnetic properties and materials

## Abstract

We investigated the effects of both intrinsic defects and hydrogen atom impurities on the magnetic properties of MgO samples. MgO in its pure defect-free state is known to be a nonmagnetic semiconductor. We employed density-functional theory and the Heyd–Scuseria–Ernzerhof (HSE) density functional. The calculated formation energy and total magnetic moment indicated that uncharged ${\mathrm{V}}_{\mathrm{Mg}}^{0}$ and singly charged ${\mathrm{V}}_{\mathrm{Mg}}^{-1}$ magnesium vacancies are more stable than oxygen vacancies (V_O_) under O-rich growth conditions and introduce a magnetic moment to MgO. The calculated density of states (DOS) results demonstrated that magnetic moments of V_Mg_ result from spin polarization of an unpaired electron of the partially occupied valence band, which is dominated by O 2p orbitals. Based on our calculations, V_Mg_ is the origin of magnetism and ferromagnetism in MgO. In contrast, the magnetic moment of the magnetic V_Mg_-MgO crystal is suppressed by hydrogen (H) atoms, and unpaired electrons are donated to the unpaired electronic states of V_Mg_ when the defect complex H_i_-V_Mg_ is formed. This suggests that H causes a reduction in magnetization of the ferromagnetic MgO. We then performed experimental studies to verify the DFT predictions by subjecting the MgO sample to a thermal treatment that creates Mg vacancies in the structure and intentionally doping the MgO sample with hydrogen atoms. We found good agreement between the DFT results and the experimental data. Our findings suggest that the ferromagnetism and diamagnetism of MgO can be controlled by heat treatment and hydrogen doping, which may find applications in magnetic sensing and switching under different environmental conditions.

## Introduction

Magnesium oxide (MgO) has shown attractive functionality in many applications, including as a material for refractory protective layers of AC plasma displays and a magnetic shielding/insulator layer in tunneling magneto-resistance (TMR) sensors for the hard disk drive (HDD) industry. MgO is a great insulator with a band gap of 7.8 eV^1,2^. It can also be synthesized in many forms by several methods^3–5^ and has been intensively studied as a barrier layer for magnetic tunnel junctions^6–10^. MgO is an important material for developing spintronic devices and has been widely utilized as a miniaturized magnetic sensor. However, there are still some features of MgO that are not understood. For example, MgO has been reported to exhibit ferromagnetism at room temperature^11,12^, while it is known to be a diamagnetic material. This makes it unsuitable for spintronic applications since the barrier is supposed to be an electrical insulator that does not interfere with the spins of other layers.

The d^0^ magnetism^13^ is one of the phenomena that cause ferromagnetism in diamagnetic materials such as MgO, HfO_2_, ZnO, and CaO. The ferromagnetism arises from metal atom vacancies in the structure and the free electrons in oxygen atoms surrounding the vacancies couple together, resulting in the unbalanced magnetic moment that generates a ferromagnetic signal in the material^12,14^. Many studies involving both calculations and experiments indicate that the strength of magnetization results from d^0^ ferromagnetism, which is related to the roles of defects and impurities in the system^15–20^. Many experiments have shown that the magnetism in MgO is induced by intrinsically charged defects, such as neutral oxygen vacancies and singly and doubly charged magnesium vacancies^11–13,17,18^. Kuang et al*.* used the PBEsol functional with density functional theory to simulate the electronic structure of MgO with vacancies and related it to the magnetic properties of the material^12^. Additionally, there have been other calculation methods leading to similar results^21–24^. This research confirmed that Mg vacancies can induce ferromagnetism in the structure. While several theoretical approaches have been used to study intrinsic cation and anion vacancies in MgO, only a few studies have been focused on ubiquitous impurities such as hydrogen. Thus, the characterization of magnetism resulting from defect complexes with different charge states formed by combination of hydrogen impurity atoms and MgO intrinsic defects is still limited. Experiments have revealed that the magnetic moment and ferromagnetism of MgO can be introduced by defect states involving cation vacancies and hydrogen. For example, the formation of oxygen vacancies upon absorption of hydrogen impurities can induce ferromagnetism in MgO nanocrystallites at room temperature^17^. Balcells et al*.* reported that the reduction of ferromagnetic properties in MgO thin films prepared by radio frequency (RF) magnetron sputtering was related to the hydrogen-driven instability of vacancy centers in the material^24^. By using X-ray absorption spectroscopy (XAS) and Fourier transform infrared spectroscopy (FTIR), our previous work showed that both the ferromagnetism and diamagnetism of MgO at room temperature can be attributed to defects involving magnesium (Mg) vacancies and oxygen-hydrogen (O–H) bonding^20^. Moreover, it was found that suppression of the ferromagnetic properties may be due to formation of O–H bonds via chemical bonding between hydrogen impurities and oxygen in the MgO structure.

Here, we perform first-principles density-functional theory calculations and experiments, such as X-ray diffraction (XRD), vibrating sample magnetometry (VSM) and Fourier transform infrared spectroscopy (FTIR), to gain a deeper understanding of the roles of hydrogen and intrinsic defects in MgO ferromagnetism based on formation of defects studied with first-principles calculation and provide a comparison between these predictions and experiments.

## Methods

### Computational details

Total energy and electronic structure calculations were performed using the spin polarized DFT approach as implemented in the Vienna ab initio simulation package (VASP)^25–27^. The projector augmented-wave method was employed^28,29^. Standard functionals such as the local density approximation (LDA) and the semilocal generalized gradient approximation (GGA) are known to underestimate band gaps and create significant uncertainties in defect energy levels and the electrical activities of defects in semiconductors, especially in wide band gap materials. Instead, hybrid functionals^30^ have emerged as a useful alternative for solving problems from underestimated band gaps and energy levels. Therefore, in this work, the exchange–correlation functional was approximated by the Heyd–Scuseria–Ernzerhof hybrid functional (HSE)^31^. For the HSE functional, a Hartree–Fock exchange mixing parameter of 0.25 with the screen parameter of 0.25 were used. The valence electrons of Mg (3s^2^3p^0^) and O (2s^2^2p^4^) wavefunctions were expanded in the plane wave basis with a cutoff energy of 500 eV. The calculated band gap of MgO was 6.42 eV, which is in agreement with the experimentally observed value of 6.3 eV^32^. We used a 64-atom supercell with a 2 × 2 × 2 repeating cubic rock-salt MgO primitive cell for defect calculations. The Brillouin zone integration was sampled using a 2 × 2 × 2 k-mesh sampling grid based on the Monkhorst–Pack scheme^33^. Relaxations were performed until the Hellman–Feynman forces acting on each atom were less than 0.01 eV/Å. The calculated lattice parameter of the bulk MgO cubic lattice was 4.199 Å, which is in agreement with our experimental value of 4.22 Å^20^.

To generate defects under different growth conditions, we calculated the formation energy of a point defect X with charge state q defined^34–37^ as

1 $${E}^{f}\left({X}^{q},{E}_{F}\right)={E}_{tot}\left({X}^{q}\right)-{E}_{tot}\left(bulk\right)-\sum_{i}{n}_{i}{\mu }_{i}+q\left({E}_{VBM}+{E}_{F}\right)$$

where ${E}_{tot}\left({X}^{q}\right)$ represents the total energy of a supercell containing a defect of type *X* in charge state *q,* while ${E}_{tot}\left(bulk\right)$ is the energy of a defect-free bulk MgO supercell. *n*_*i*_ is the number of atoms from species *i* (Mg, O, or H) added to a supercell to form the defect cell. μ_i_ is the atomic chemical potential of atomic species *i*, E_VBM_ is the valence band maximum (VBM) of MgO and E_F_ is the electron Fermi energy. The valence band energy alignment between defect and defect-free supercell approaches was used^38,39^. In Eq. (1), ${E}_{VBM}$ refers to the valence band maximum (VBM) and represents the electron chemical potential reference. However, the Fermi energy is zero in the DFT calculation, and the absolute value is meaningless for a periodic system. The position of the VBM of a supercell containing a defect is different from that of a defect-free supercell, and this difference depends, in general, on the charge state of the defect. Therefore, a valence band alignment correction is usually applied by matching the two values. The magnitude of this correction is ∆V =  < V_bulk_ > − < V_defect_ > , where < V_bulk_ > and < V_defect_ > are the average self-consistent potentials calculated for the bulk and defect supercells, respectively. The alignment correction term (∆V) was approximately 0.1–0.4 eV for our calculations. The chemical potentials μ_Mg_, μ_O_, and μ_H_ were limited by the energies of solid Mg, gaseous O_2_, and H_2_, which we referenced as zero energy. To grow MgO in thermal equilibrium, the chemical potential should satisfy ${\mu }_{Mg}+{\mu }_{O}={\mu }_{MgO}.$ The calculated MgO chemical potential is ${\mu }_{MgO}=-6.26 \mathrm{eV}$, which is in agreement with the experimental enthalpy of formation value of − 6.236 eV (− 601.7 kJ/mol)^39^. Therefore, in our calculations, we used ${\mu }_{Mg}=-6.26 \mathrm{eV}-{\mu }_{O}$, where − 6.26 ⩽ ${\mu }_{O}$ ⩽0 (note that ${\mu }_{O}=0$ was defined as half of the O_2_ energy). In the presence of O, Mg forms MgO rather than solid Mg. Therefore, the upper limit of ${\mu }_{Mg}$ is set by MgO precipitation limits, i.e., ${\mu }_{Mg}^{max}$= ${\mu }_{Mg}={\mu }_{MgO}$-${\mu }_{O}$. For oxygen-rich conditions (${\mu }_{O}=0$), ${\mu }_{Mg}^{max}$= − 4.06 eV.

### Experimental procedure

The MgO samples used in this study contained defects such as Mg vacancies (V_Mg_) and hydrogen atom impurities that were intentionally introduced into the samples. We note that all MgO samples were prepared from 96.0% MgO commercial powder (LOBA Chemie). V_Mg_ was induced into the commercial MgO powder by heating it in a vacuum (pressure of 2 × 10^–2^ Torr) at a temperature of 550 °C, and this was labeled “MgO vacuum heated”. After that, intentional hydrogen doping was carried out by flowing ultrahigh purity hydrogen gas (99.999%) at room temperature to the vacuum heated MgO sample for at least 5 h with different pressures, including 40 and 70 bars; these samples were labeled MgO vacuum heated with H_2_ gas doping of 40 and 70 bars, respectively. Their magnetic behaviors were measured with a vibrating sample magnetometer (VSM) option in the VersaLab instrument (Quantum Design, USA) operated at applied magnetic fields between − 5000 and 5000 Oe. The structures of all MgO samples were investigated with X-ray diffraction (XRD, Bruker D8 ADVANCE) using Cu-Kα radiation and operated at 40 kV and 40 mA. For Rietveld refinement analyses, high-quality X-ray diffraction patterns were collected in the 2-theta range 10°–120° with an interval step of 0.02° and a scan rate of 2 s/step. Rietveld refinement, using TOPAS software, was utilized to obtain crystallographic information. The FP peak type was utilized as the peak profile for fitting. Note that KCl was inserted into the XRD samples to act as an internal standard. The presence of the hydrogen dopant was characterized by Fourier transform infrared spectrometry (FT-IR, Bruker Tensor 27-Hyperion).

## Results and discussion

### Defect formation energy and electronic structures

**Figure 1:**
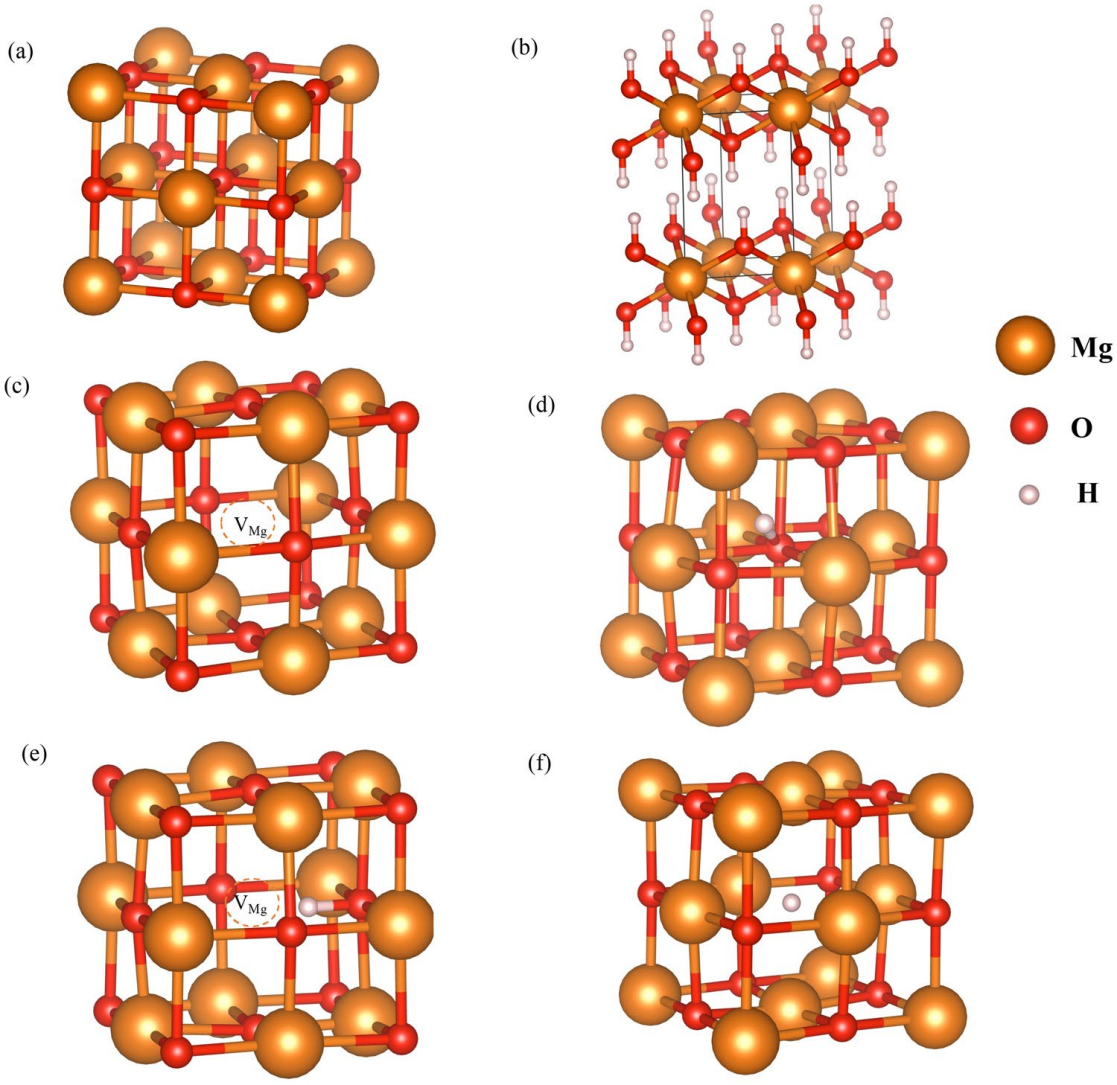
Relaxed atomic structural models of (**a**) free defect MgO bulk, (**b**) Mg(OH)_2_ bulk, (**c**) ${\mathrm{V}}_{\mathrm{Mg}}^{0}$, (**d**) H_i_^+1^, (**e**) (H_i_-V_Mg_)^−1^ and (**f**) H_O_^+1^.

As a first step toward understanding the magnetic properties and effects of hydrogen impurity atoms in MgO, we obtained the electronic structures and formation energies of intrinsic O and Mg vacancy defects as well as H impurity atoms. It was found in previous studies that when the aforementioned defects were present in MgO, they affected the magnetic properties of MgO^17,24,41,42^. Figure 1 shows relaxed atomic structural models for perfect bulk MgO (Fig. 1a) and the hydroxide salt Mg(OH)_2_ (Fig. 1b), which was used as a reference and to study the hydrogen formed in MgO compounds in nature. The relaxed atomic structure of the point defect form (such as ${\mathrm{V}}_{\mathrm{Mg}}^{0}$, H_i_^+1^, (H_i_-V_Mg_)^−1^ and H_O_^+1^), which focuses on nearest neighbor atoms, is also shown in Fig. 1c–f, respectively. The perfect bulk MgO has a rock salt structure with a lattice constant of 4.199 Å, while the hydroxide form Mg(OH)_2_ is hexagonal with lattice constants a = b = 3.186 Å and c = 4.782 Å; however, the Mg atoms in both structures are surrounded by six oxygen atoms in an octahedral configuration such that the Mg-O bond lengths in MgO and Mg(OH)_2_ are 2.099 Å and 2.115 Å, respectively. We found that the lowest energy atomic configuration of interstitial hydrogen in MgO (Fig. 1d) was quite similar to that of H formed in Mg(OH)_2_. The interstitial H was bonded to an oxygen atom and bent off-axis from the Mg-O bond direction with an O–H bond length of 0.988 Å, which is comparable with the O–H bond length of 0.972 Å seen for Mg(OH)_2_. For the V_Mg_ structure (Fig. 1b), the O anion moved away from the point defect; on the other hand, the Mg cation moved toward the point defect, causing the total volume of the cell around the point defect to decrease because Mg is larger than O. In the H_i_ case (Fig. 1d), the O nearest neighbor H atoms moved toward the H interstitial atom, whereas Mg moved outward from H_i_ because of the Coulomb repulsion between the cations, which increased the total volume of the cell. However, when the V_Mg_ and H_i_ form H_i_-V_Mg_ complexes (Fig. 1e), the O anion still moves away from the V_Mg_ point defect, and Mg moves outward from H_i_ but less than in the previous single point defect cases because Coulombic repulsions were suppressed, resulting in a decrease in the total volume of the cell. Note that in calculations of defect cases, we found an approximate lattice constant by measuring the average Mg-to-Mg distance for atoms that surround the defect point (as shown in Fig. 1). The calculated lattice constants for ${\mathrm{V}}_{\mathrm{Mg}}^{0}$, H_i_^+1^, (H_i_-V_Mg_)^0^ and (H_i_-V_Mg_)^−1^ were 4.142, 4.422, 4.103, and 4.093 Å, respectively.

**Figure 2:**
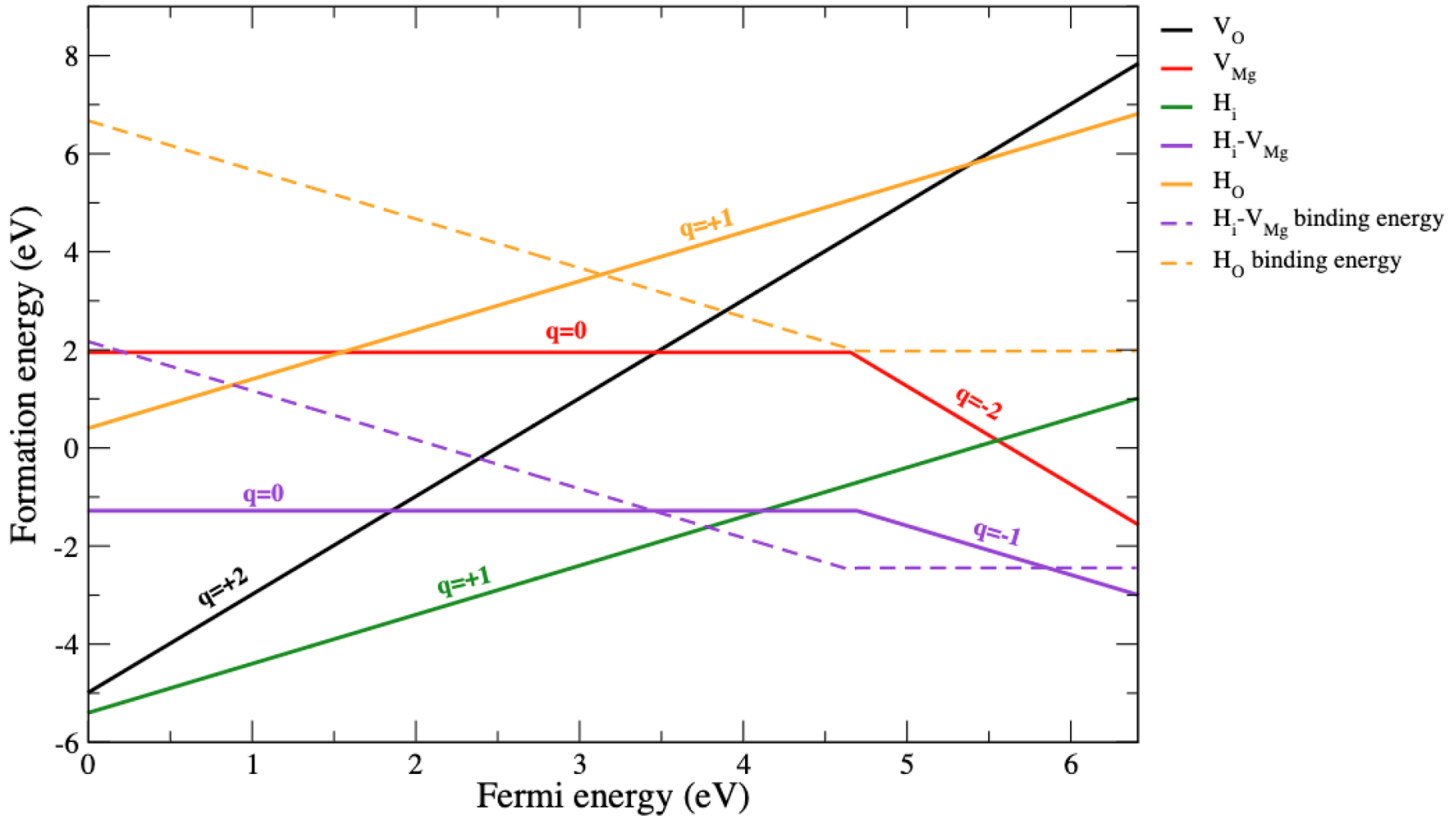
Formation energies of defects (solid lines) in MgO as a function of Fermi energy under O-rich growth conditions. Only the formation energy for the lowest energy charge state is shown. The range of Fermi energies is limited by the calculated band gap of bulk MgO, the zero Fermi energy is a relative value with the valence band maximum energy, and the maximum Fermi energy represents the conduction band minimum. The slopes of the plots reflect changes in the charge states of the defect, and the kinks in the plots correspond to the energies at which transition from one charge state to another takes place, as discussed in the text. The binding energy levels of the (H_i_-V_Mg_) complex and H_O_ are plotted as a function of Fermi energy with dashed lines.

Figure 2 shows the formation energies calculated under O-rich conditions for the oxygen vacancy (V_O_) (solid black line), magnesium vacancy (V_Mg_) (solid red line), hydrogen impurity (H_i_) (solid green line), hydrogen impurity formed with a Mg vacancy (H_i_-V_Mg_) (solid purple line), and hydrogen impurity substituted on oxygen vacancy complex (H_O_) (solid yellow line) plotted as a function of Fermi energy. The zero Fermi energy is relative to the valence band maximum level, while the maximum Fermi energy represents the conduction band minimum. The slope of each line indicated the change in the charge state of defect *q* (Eq. 1), and only the most stable charged states were shown.

For the formation energies of native vacancies (Fig. 2), V_O_ is a donor defect with the lowest formation energy at a Fermi energy below 3.5 eV (p-type growth condition), which can be stable with + 2 charges and form with a negative formation energy at Fermi energies below 2.5 eV. V_Mg_ is the lowest formation energy at a Fermi energy above 3.5 eV (n-type growth condition) and forms with a negative value at a Fermi energy above 5 eV. V_Mg_ is a shallow acceptor with a defect transition level change from the charge state q = 0 to the charge state q = − 2 (ɛ(0/− 2)) at a Fermi energy level of 4.7 eV. The relationship between the formation energy of a defect *X* of charge state *q*, ${E}^{f}\left({X}^{q}\right),$ and its concentration $C\left({X}^{q}\right)$ for a sample under thermal equilibrium is defined as $C\left({X}^{q}\right)={N}_{site}exp[{-E}^{f}\left({X}^{q}\right)/{k}_{B}T]$ ^40^, where ${N}_{site}$ is the number of equivalent possibilities with the same energies in which the defect can be incorporated (per unit volume), ${k}_{B}$ is Boltzmann’s constant, and *T* is the temperature. Therefore, the defects with the lowest formation energies are stable and likely to form at high concentrations. These results suggest that V_O_ and V_Mg_ can occur in the Fermi energy range of the sample under O-rich growth conditions because both defects can be formed with highly negative formation energies. In addition, the defect concentration can be increased by increasing the temperature (*T*) of the sample. In general, the shift in Fermi energy due to doping is proportional to the temperature^35^_._ If a MgO sample is heated at high temperature, the Fermi level will shift to a high energy. Therefore, under heat treatment conditions, V_Mg_ is more likely to form than V_O._ When hydrogen (H) is doped into the MgO system, among several possible configurations studied for H_i_, the interstitial structure shown in Fig. 1d turned out to have the lowest energy. The H impurity is likely to occupy interstitial sites close to an oxygen atom and act as a donor defect with + 1 charge and formation energy lower than both V_O_ and V_Mg_. As a charged defect, it can be compensated by a native defect with the opposite charge. Potential compensating point acceptor defects include V_Mg_. The formation energy of the H_i_ complex formed with V_Mg_ (H_i_-V_Mg_) was calculated (the atomic structure is shown in Fig. 1e), and H_i_-V_Mg_ was stable for charge states 0 (H_i_-V_Mg_)^0^ and -1 (H_i_-V_Mg_)^−1^ (Fig. 2). H_i_^+1^ can donate an electron to the available states of V_Mg_ charge state 0 (${\mathrm{V}}_{\mathrm{Mg}}^{0}$) and -2 (${\mathrm{V}}_{\mathrm{Mg}}^{-2}$), respectively. H_i_-V_Mg_ is a shallow acceptor exhibiting a defect transition level change from charge state q = 0 to charge state q = -1 at a Fermi energy level of 4.7 eV with a negative formation energy over the entire Fermi level range. This indicated that H_i_-V_Mg_ is quite stable and can form at high concentrations. Therefore, V_Mg_ can be a compensating defect in H-doped MgO samples and form H_i_-V_Mg_. In the case of V_O_, hydrogen impurities can form defect complexes with V_O_ by substitution at vacancy sites surrounded by Mg cations (the atomic structure is shown in Fig. 1f). The H_O_ acts as a shallow donor, and it is stable only in charge state + 1 (Fig. 2), meaning that the H acts as H^−1^ to react with V_O_ in a stable charge state + 2 (V_O_^2+^). Therefore, the H in H_O_ behaves as a negative multicenter for defect structures, in agreement with Janotti et al*.*^43^. However, the formation energy of H_O_ is relatively high and positive throughout the Fermi level range for O-rich conditions. To determine the stabilities of various defect complexes (i.e., Fig. 2), we obtained the defect binding energy, which may be defined as *E*_*bind*_* (A-B)* = *E*_*f*_* (A-B) − (E*_*f*_* (A)* + *E*_*f*_* (B)),* where *E*_*f*_* (A)* and *E*_*f*_* (B)* are the formation energies of defects A and B, respectively, and *E*_*f*_* (A-B)* is the formation energy of the A–B complex^44^. The complex had a large negative binding energy and is more likely to form. In Fig. 2, we observe the lowest binding energy of − 2.45 eV (purple dashed line) for H_i_-V_Mg_ and 1.97 eV (yellow dashed line) for the H_O_ complex. The binding energy of H_i_-V_Mg_ becomes negative at a high Fermi level (> 4.5 eV) close to the conduction band energy. This indicates that such complex defects are most stable and likely to form at a high concentration in that energy region. However, H_i_-V_Mg_ is the dominant defect complex and has a lower binding energy than the H_O_ complex under O-rich growth conditions. This indicates that H_i_ can interact strongly with V_Mg_ in MgO, which might cause suppression of the ferromagnetic properties of MgO, as reported in other experimental studies^20,24^.

**Figure 3:**
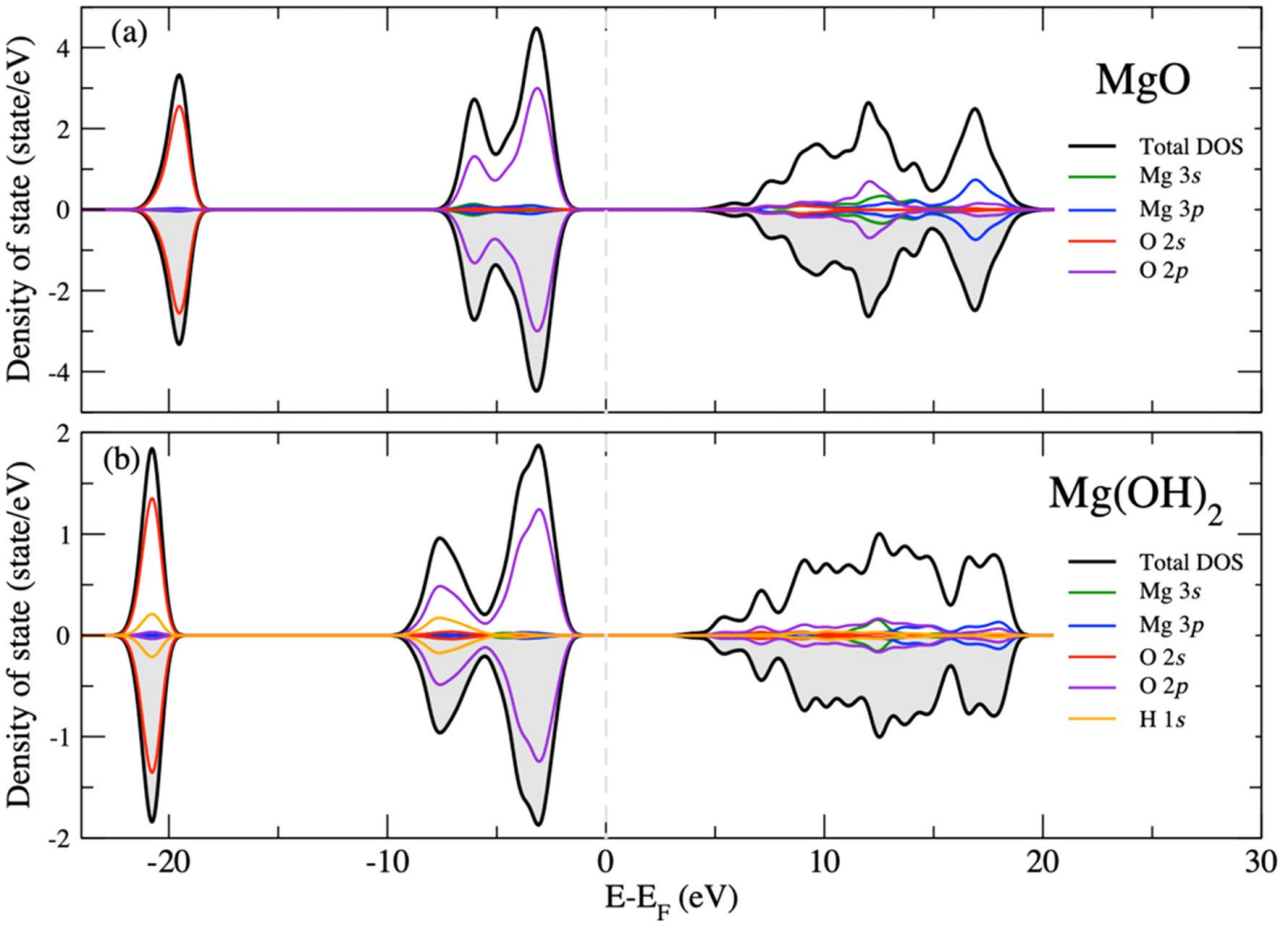
Total density of states and projected density of states for (**a**) MgO and (**b**) Mg(OH)_2_ with projected orbitals (*s*, *p,* and *d* orbitals) from each atom. The Fermi energy is shifted to zero and is indicated by the gray dashed line. The white and gray shaded areas represent spin-up and spin-down electronic densities of states, respectively.

**Figure 4:**
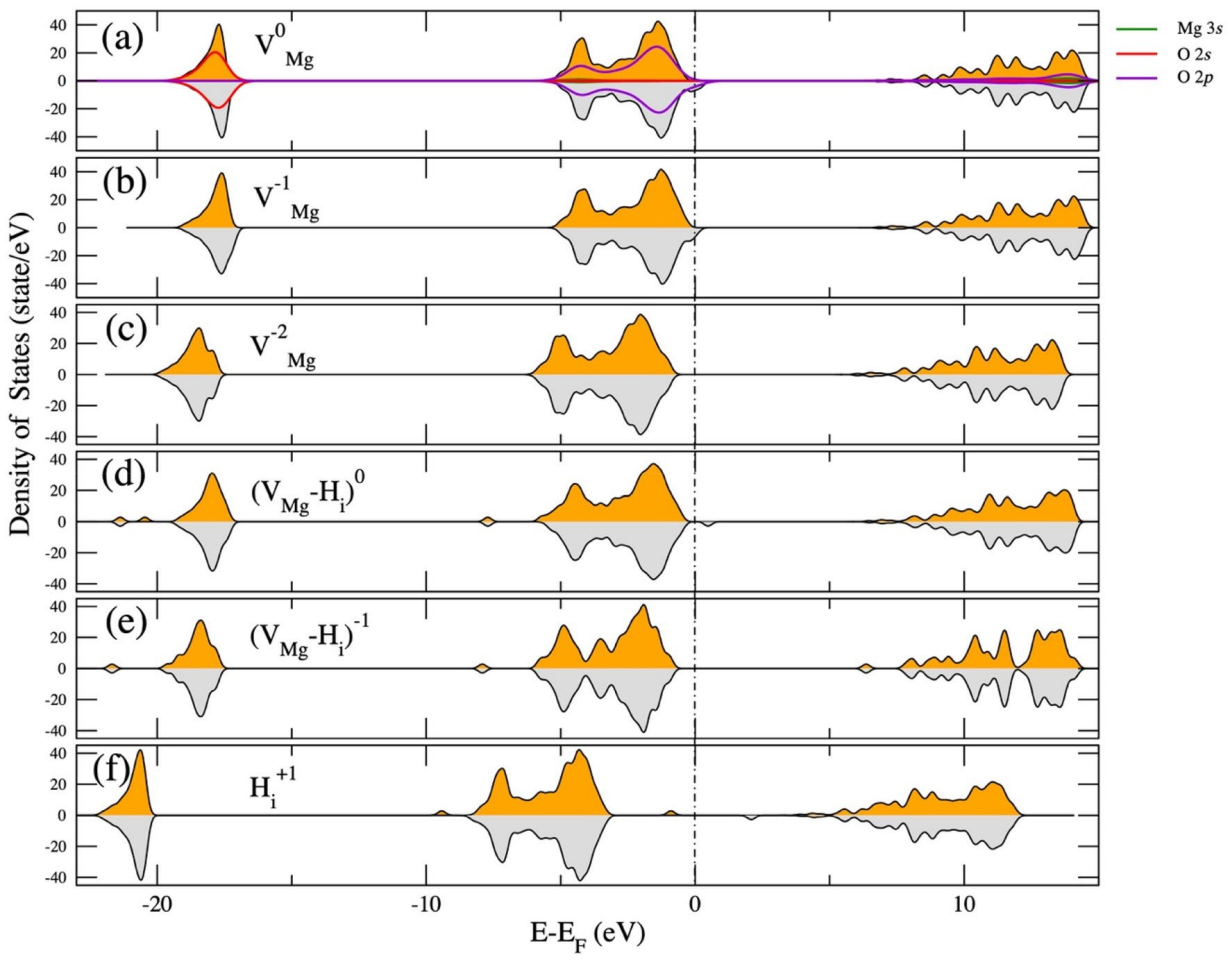
Total density of states for various defects in MgO with (**a**) neutral ${\mathrm{V}}_{\mathrm{Mg}}^{0}$ with projected atomic orbitals, (**b**) singly charged ${\mathrm{V}}_{\mathrm{Mg}}^{-1}$, (**c**) doubly charged ${\mathrm{V}}_{\mathrm{Mg}}^{-2}$, (**d**) neutral ${\left({\mathrm{H}}_{\mathrm{i}}-{V}_{Mg}\right)}^{0}$ complex, (**e**) singly charge $\mathrm{d }{ ({\mathrm{H}}_{\mathrm{i}}-{V}_{Mg})}^{-1}$ and (**f**) singly charged ${\mathrm{H}}_{\mathrm{i}}^{+1}$. The orange and gray shaded areas represent spin-up and spin-down electronic densities of states, respectively. The Fermi energy is shifted to zero and indicated by a black dash-dotted line.

To understand the origin of defect-induced magnetization in MgO, we calculated the density of states (DOS) for perfect MgO and Mg(OH)_2_ (Fig. 3), as well as for various defect types (Fig. 4). The valence was defined by the state below the Fermi level. According to the orbital-resolved projected DOS, the valence bands of both perfect MgO (Fig. 3a) and Mg(OH)_2_ (Fig. 3b) were dominated by O 2 s (red) and O 2p (violet) orbitals, whereas almost all Mg 3 s (green) and 3p (blue) orbitals contributed to the conduction band. The valence band maximum was occupied only by O 2p electrons. In comparison, the H 1 s orbitals (orange line in Fig. 3b) contributed to the upper (energy below − 5 eV) and lower valence bands (energy below − 20 eV for Mg(OH)_2_), and the H 1 s orbitals formed covalent bonds with both O 2 s and O 2p orbitals. We found that the DOS for perfect MgO and Mg(OH)_2_ were symmetrical over the entire energy range for both spin-up (white area) and spin-down (gray area) states, indicating that the sums of the magnetic moments or magnetizations of both MgO and Mg(OH)_2_ were zero, in good agreement with other published works^12,22,23^. In other words, neither MgO nor Mg(OH)_2_ have any unpaired electron states. Therefore, a magnetic moment cannot occur in pure MgO or Mg(OH)_2_.

The total magnetic moments and specific magnetizations of various defect types are summarized in Table 1. The magnetic moment due only to the spin of an electron is 1 Bohr Magneton (*µ*_B_) = 9.27 × 10^–21^ emu. To convert this into magnetization, which is the magnetic moment per gram (or the magnetic moment per unit volume), we used the magnetic moment per cubic cell and divided this moment by the cell volume to obtain the magnetization in units of emu/cm^3^. The specific magnetization (emu/g) was obtained by dividing the above magnetization by the MgO density (3.47 g/cm^3^). Our calculations showed that neutrally charged ${\mathrm{V}}_{\mathrm{Mg}}^{0}$ and singly charged ${\mathrm{V}}_{\mathrm{Mg}}^{-1}$ and ${\mathrm{V}}_{\mathrm{O}}^{+1}$ were responsible for the magnetic moment of MgO, which originates from the partially occupied valence band. Other defects, i.e., ${\mathrm{V}}_{\mathrm{Mg}}^{-2}$, ${\mathrm{V}}_{\mathrm{O}}^{0}$, and ${\mathrm{V}}_{\mathrm{O}}^{+2}$, cannot induce magnetic moments in MgO. However, according to the formation energy, binding energy and magnetic moment results, oxygen vacancies can be ruled out under our conditions, as discussed above. In addition, there is ${\mathrm{V}}_{\mathrm{O}}^{+1}$, which is responsible for the magnetic moment, but ${\mathrm{V}}_{\mathrm{O}}^{+1}$ is not the charge defect with the lowest formation energy.

**Table 1:** Total magnetic moments (M) in Bohr magnetons (*µ*_B_) and the specific magnetization in emu/g within corresponding defects.

Defect	M (*µ*_B_)	Magnetization (emu/g)
${\mathrm{V}}_{\mathrm{Mg}}^{0}$	2.04	9.75
${\mathrm{V}}_{\mathrm{Mg}}^{-1}$	1.00	4.81
${\mathrm{V}}_{\mathrm{Mg}}^{-2}$	0.00	0.00
${\mathrm{V}}_{\mathrm{O}}^{0}$	0.00	0.00
${\mathrm{V}}_{\mathrm{O}}^{+1}$	1.00	4.51
${\mathrm{V}}_{\mathrm{O}}^{+2}$	0.00	0.00
${\mathrm{H}}_{i}^{+1}$	1.00	3.86
${({\mathrm{H}}_{\mathrm{i}}-{\mathrm{V}}_{\mathrm{Mg}})}^{0}$	− 1.00	4.84
${({\mathrm{H}}_{\mathrm{i}}-{\mathrm{V}}_{\mathrm{Mg}})}^{-1}$	0.00	0.00
${({\mathrm{H}}_{\mathrm{i}}-{\mathrm{V}}_{\mathrm{Mg}})}^{+1}$	0.00	0.00
${\mathrm{H}}_{\mathrm{O}}^{0}$	1.00	4.10
${\mathrm{H}}_{\mathrm{O}}^{+1}$	0.00	0.00

Figure 4a shows the total density of states for neutral ${\mathrm{V}}_{\mathrm{Mg}}^{0}$, which creates the hole state in MgO. Spin splitting exists in the valence band, and the spin-down states (gray area) are partially occupied, which is the cause for formation of the magnetic moment of ~ 2 *µ*_B_. The projected atomic orbital DOS shows that the maximum valence is dominated by O 2p orbitals (solid violet line), which indicates that the magnetic moment is mainly induced by O 2p orbitals. In the same way, for ${\mathrm{V}}_{\mathrm{Mg}}^{-1}$ (Fig. 4b), spin splitting also occurs in the valence band, and the spin-down states (gray area) are partially occupied; hence, ${\mathrm{V}}_{\mathrm{Mg}}^{-1}$ also creates a magnetic moment (~ 1 *µ*_B_). In comparison, the spin-polarized electron orbitals of ${\mathrm{V}}_{\mathrm{Mg}}^{-2}$ are symmetric. Therefore, ${\mathrm{V}}_{\mathrm{Mg}}^{-2}$ cannot induce a magnetic moment (Fig. 4c). In other words, when removing Mg from MgO, the Mg-O bond is broken to form ${\mathrm{V}}_{\mathrm{Mg}}^{0}$ or ${\mathrm{V}}_{\mathrm{Mg}}^{-1}$, which lose two and one donor electrons. This mechanism induced the hole state from O 2*p* orbitals to compensate for the loss of electrons. However, ${\mathrm{V}}_{\mathrm{Mg}}^{0}$ has the lowest formation energy and can induce a magnetic moment. Therefore, under our experimental conditions, ${\mathrm{V}}_{\mathrm{Mg}}^{0}$ is the origin of the magnetism in MgO.

When we dope a donor H impurity into perfect MgO (H_i_), the H_i_ defect creates unpaired states below the Fermi level and below the valence band (Fig. 4f) with H 1 s orbitals. This doping introduces a magnetic moment of 1μ_B_ (see Fig. 4f) because H_i_ contributes one 1 s electron to the valence band (Fig. 4f). Hence, when the H impurity atom forms a defect complex with ${\mathrm{V}}_{\mathrm{Mg}}^{0}$ such as (${\mathrm{H}}_{\mathrm{i}}-{\mathrm{V}}_{\mathrm{Mg}})$, the H donates an electron to the O 2p hole state in the valence band of MgO, which is created by ${\mathrm{V}}_{\mathrm{Mg}}$ and fills the unpaired state, then the magnetic moments remain 1 and 0 for ${({\mathrm{H}}_{\mathrm{i}}-{\mathrm{V}}_{\mathrm{Mg}})}^{0}$ and ${({\mathrm{H}}_{\mathrm{i}}-{\mathrm{V}}_{\mathrm{Mg}})}^{-1}$, respectively (see the DOS in Fig. 4d,e). Based on our calculation results, the origin of the magnetic moment in MgO is largely an unpaired electron from a Mg vacancy, in agreement with other works^12,21–24^. Hydrogen impurities suppress the magnetic moment in magnetic MgO by donating electrons to unpaired electronic states. Therefore, hydrogen is the most significant cause of the reduced magnetic properties in ferromagnetic MgO, which might occur in aged MgO samples^20,24^. For example, magnetic MgO samples are treated in air or under hydrogen gas. In this work, the V_Mg_ in MgO was induced by thermal heating in a vacuum environment because V_Mg_ is easy to form at low energy, whereas H_2_ gas injection into the MgO samples at different pressures was used to represent hydrogen doping. The structural and magnetic properties of MgO will be presented and discussed in the Experimental Section.

### Experimental results

**Figure 5:**
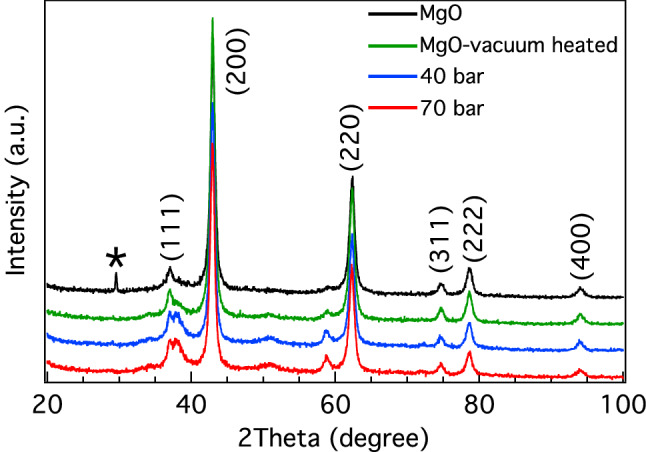
XRD patterns for 96% commercial MgO (black line), MgO vacuum heated (green line), and MgO vacuum heated with H_2_ gas doping at 40 bar (blue line) and 70 bar (red line). * is marked as CaCO_3_ impurity.

Figure 5 shows the XRD patterns for 96% MgO commercial powder (black line), vacuum heated (green line) MgO, and MgO vacuum heated with H_2_ gas doping at 40 bar (blue line) and 70 bar (red line). The diffraction peaks of the starting material were only indexed as the rock salt structure (Fm-3 m space group) of MgO, as indicated by ICDD No. 01-076-6597. The phase analyses for all samples mainly indicated MgO compositions. A small amount of CaCO_3_ is detected at 30° in the MgO commercial powder (black line), but this peak disappeared after heat treatment. Following the heat treatment, the data for the crystals were in agreement with ICDD No. 01-076-6597. We observed new small peaks present at approximately 35°, 50°, and 60° for the vacuum heated MgO sample (green line). Moreover, all of the new small peaks were significantly stronger after H_2_ gas doping at 40 and 70 bar (blue and red lines) and are possibly related to vacancy defects, H impurities, crystal direction, and lattice distortions in the MgO structure.

To further characterize the XRD results, Rietveld refinements of the data for the 96% MgO commercial sample and the vacuum heated MgO sample were utilized to obtain crystallographic information. Rietveld refinement is a useful method for investigating the full crystal structure details of synthesized materials, e.g., lattice parameters, atomic coordinates, and atomic occupancies. The approach is based on the least squares method, which can be described with the equation: ${S}_{y}= \sum_{i}{w}_{i}[{y}_{i\left(obs\right)}-{y}_{i(calc)}{]}^{2}$, where ${w}_{i}$ = $1/ {y}_{i}$, ${y}_{i(obs)}$ is the observed intensity, ${y}_{i(calc)}$ is the calculated intensity and ${S}_{y}$ is the weighted difference between these two values at step *i*. The refinement procedure involves fitting of the observed XRD pattern to the calculated model structure or crystallographic information file to achieve the minimum ${S}_{y}$. The fitting can be evaluated by the weight-profile R-value or ${R}_{wp}$, where ${R}_{wp}= {\left\{{\sum_{i}{w}_{i}\left[{y}_{i}\left(obs\right)-{y}_{i}(calc)\right]}^{2}/\sum_{i}{w}_{i}{\left[{y}_{i}(obs)\right]}^{2}\right\}}^{1/2}$. The site occupancy factor (SOF) is used to determine the fraction of a site occupied by a specific atom, which can be obtained with the structural factor (${F}_{hkl}$) equation: ${F}_{hkl}= \sum_{j=1}^{m}{N}_{j}{f}_{j}exp\left[2\pi i({hx}_{j}+{ky}_{j}+{lz}_{j})\right]$, where ${f}_{j}$ is the scattering factor, ${N}_{j}$ is the SOF, (hkl) is the atomic plane and (x, y, z) are fractional coordinates. If the SOF is equal to 1, every equivalent position (x, y, z) is occupied. However, some of the sites are vacant if the SOF is less than 1.

**Figure 6:**
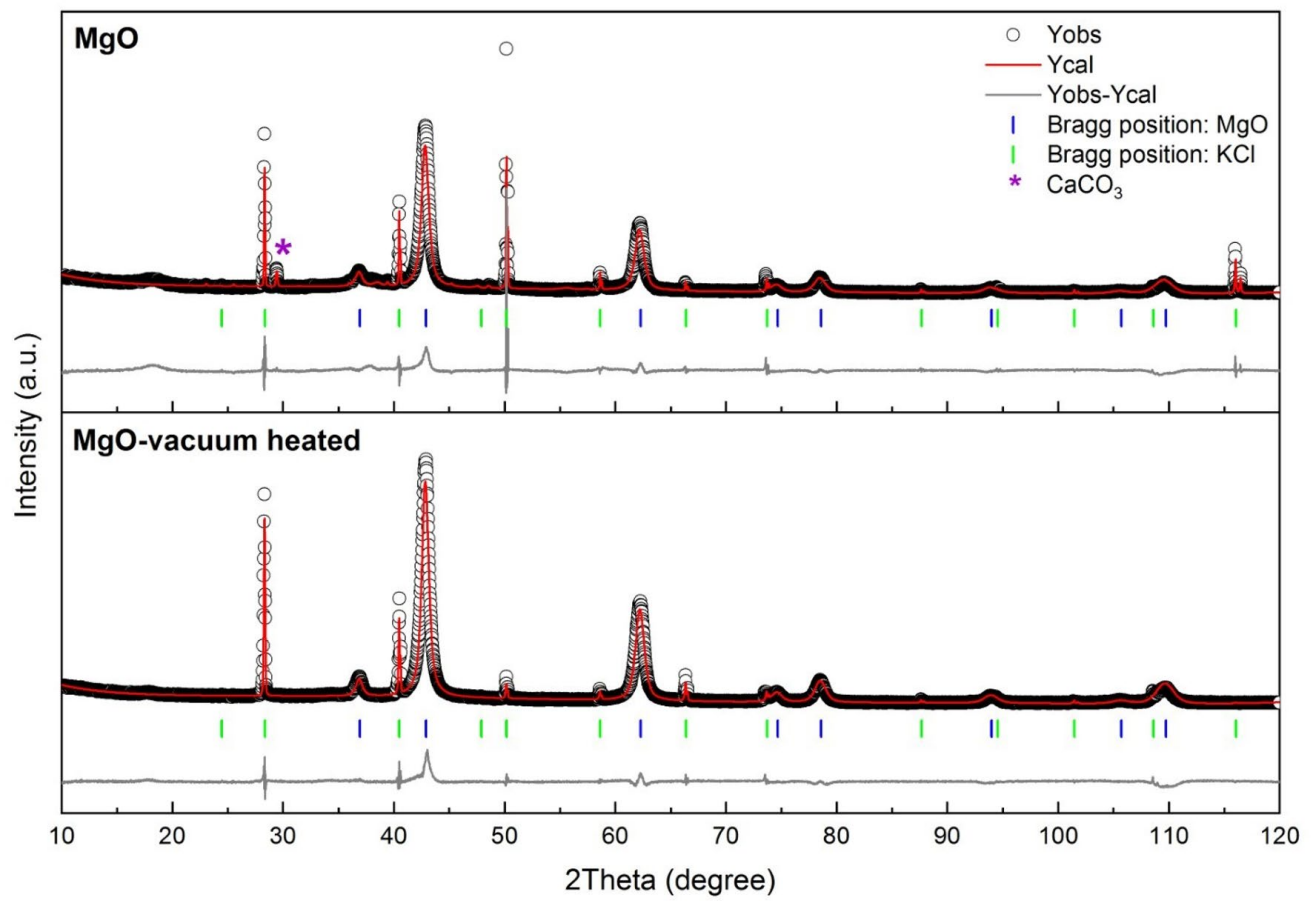
Rietveld refinement plots of MgO and MgO-vacuum heated samples. MgO crystallized in the Fm-3 m (225) space group. KCl was used as an internal standard representing the CaCO_3_ phase.

Figure 6 shows Rietveld refinement plots for the 96% MgO commercial sample and the MgO vacuum heated sample. In this work, the fits between the observed XRD patterns and the structural model showed convergence with good weight-profile R values (%R_wp_) and goodness of fit (GOF), as shown in Table 2. The cell parameter *a* was decreased from 4.2202(2) Å to 4.2163(1) Å upon vacuum heating, which agreed with our DFT relaxed structure. The V_Mg_ results in a decrease in the lattice constant of the cell. The calculated and experimental lattice constants are summarized in Table 3. The results also revealed the phase compositions for both samples; the MgO sample contained 97.79(18) wt% of the MgO phase and 2.21(18) wt% of CaCO_3,_ while the MgO-vacuum heated sample included only the MgO phase. The crystallographic details obtained are shown in Tables 2 and 4. The site occupancy factor (SOF) was determined to be 1.0000(56) for Mg in the MgO sample, corresponding to the stoichiometric ratio in the starting material. However, the value was reduced to 0.9623(31) after vacuum heating, which indicated the formation of Mg vacancies. This result corresponded to the formation energy predicted by DFT, and Mg vacancies were induced by vacuum heating and are more stable than oxygen vacancies. Furthermore, equivalent isotropic thermal parameters (Beq.) of Mg and O atoms in MgO 96% commercial powder and MgO-vacuum heated samples corresponded to the crystallographic information file (CIF file), as shown in Table 4. These values are useful for describing the motions of atoms in crystal structures, where lighter atoms vibrate stronger and exhibit higher values.

**Table 2:** Crystallographic information obtained from Rietveld refinement of data for MgO and MgO vacuum heated samples (Mg-Vac)”.

Sample	Residual parameters				Lattice parameters	
	R_exp_ (%)	R_p_ (%)	R_wp_ (%)	GOF	*a* (Å)	*V* (Å^3^)
MgO	3.05	9.47	12.37	4.06	4.2202 (2)	75.16 (1)
MgO-Vac	2.85	6.40	8.66	3.04	4.2163 (1)	74.95 (1)

**Table 3:** Experimental lattice constants for various experimental samples determined from XRD spectra (Fig. 5) and calculated lattice constants from predicted cases.

Samples	Experimental lattice constant (Å)	Calculated lattice constant (Å)
MgO-commercial powder	4.22	4.20
MgO-vacuum heated	4.21	4.14
MgO vacuum heated with H_2_ gas dopant at 40 bars	4.21	4.10
MgO vacuum heated with H_2_ gas dopant at 70 bars	4.21	4.09

**Table 4:** Atomic coordinates, site occupancy factors (SOF) and equivalent isotropic thermal parameters (B_eq._) of MgO in MgO and MgO-vacuum heated samples.

Atom	Wyckoff Site	*x*	*y*	*z*	SOF	B_eq_
**MgO**						
Mg	4*a*	0	0	0	1.0000 (56)	0.312
O	4*b*	1/2	1/2	1/2	1	0.362
**MgO-vacuum heated**						
Mg	4*a*	0	0	0	0.9623 (31)	0.312
O	4*b*	1/2	1/2	1/2	1	0.362

**Figure 7:**
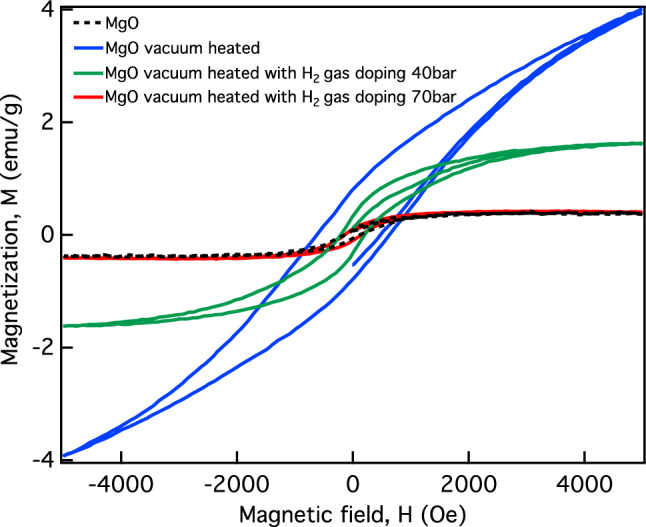
Magnetization-magnetic field hysteresis curves for MgO (black dashed line), vacuum heated MgO (solid blue line), MgO vacuum heated with H_2_ gas doping at 40 bar (solid green line), and MgO vacuum heated with H_2_ gas doping at 70 bar (solid red line).

Figure 7 shows the magnetization-magnetic field hysteresis curves (M-H hysteresis curves) of commercial MgO powder (black dashed line), vacuum heated MgO (solid blue line), and MgO vacuum heated with H_2_ gas at 40 bar (solid green line) and 70 bar (solid red line). Note that all presented data were analyzed with subtraction of the diamagnetic components. After subtraction, we found that the M-H hysteresis curves of all MgO samples showed ferromagnetic behavior with different magnetization values. The 96.0% MgO commercial powder produced an amplitude for the M-H curve with a small magnetization value of approximately 0.31 emu/g, which is close to zero. According to the DFT prediction, bulk MgO is diamagnetic, which should result in a magnetization value of 0 emu/g. In addition, the SOF values for 96.0% MgO commercial powder (Table 4) were equal to 1 for both Mg and O atoms. Therefore, the small magnetism of the commercial powder was from some magnetic element contaminant, and the specification data for commercial MgO powder included 0.0025% Fe and 0.0005% Pb. After heat treatment, the M-H hysteresis curve of the MgO vacuum heated sample increased to give the highest ferromagnetic magnetism of approximately 3.9 emu/g. We propose that the V_Mg_ induced by heat treatment caused the increase in the ferromagnetic magnetism of the MgO vacuum heated sample. Another possible mechanism for inducing magnetism may involve converting H impurities back to their original state (V_Mg_) by using heat treatment^20,45^. Next, by doping with hydrogen, we found that the ferromagnetic magnetism of MgO vacuum heated with H_2_ gas decreased to approximately 1.63 and 0.31 emu/g for gas pressures of 40 and 70 bars, respectively. Based on our theoretical DFT prediction, Table 1 shows that the neutral ${\mathrm{V}}_{\mathrm{Mg}}^{0}$, which was created by vacuum heating, has a total magnetic moment of 2.0478 *µ*_B_ or 9.75 emu/g, corresponding to a concentration of 3.06% for Mg vacancies (one Mg vacancy in a 64-atom supercell). If we dope hydrogen with the same concentration as V_Mg_, hydrogen will form a ${({\mathrm{H}}_{\mathrm{i}}-{\mathrm{V}}_{\mathrm{Mg}})}^{0}$ complex that gives a total magnetic moment of 1*µ*_B_ or 4.83 emu/g. This prediction corresponds with experiment; when we treated the MgO vacuum heated sample with H_2_ gas at 40 bar, the maximum ferromagnetic magnetism dropped from 3.9 to 1.63 emu/g, which was approximately 50% lower than the DFT prediction. Next, if we continued to treat the vacuum heated MgO with a hydrogen concentration greater than the V_Mg_ concentration, the situation might be described as ${({\mathrm{H}}_{\mathrm{i}}-{\mathrm{V}}_{\mathrm{Mg}})}^{-1}$ cases. The magnetization of ${({\mathrm{H}}_{\mathrm{i}}-{\mathrm{V}}_{\mathrm{Mg}})}^{-1}$ will be reduced to zero, consistent with DFT prediction, as shown in Table 1. This is because hydrogen donates an electron to ${({\mathrm{H}}_{\mathrm{i}}-{\mathrm{V}}_{\mathrm{Mg}})}^{0}$, which completely fills the unpaired state of ${({\mathrm{H}}_{\mathrm{i}}-{\mathrm{V}}_{\mathrm{Mg}})}^{0}$. This results in conversion of the magnetization for the vacuum heated MgO treated with H_2_ gas at 70 bar back to the MgO initial value. We showed how introduction of magnesium vacancies V_Mg_ and hydrogen atom impurities can be used to control the magnetic properties of MgO.

**Figure 8:**
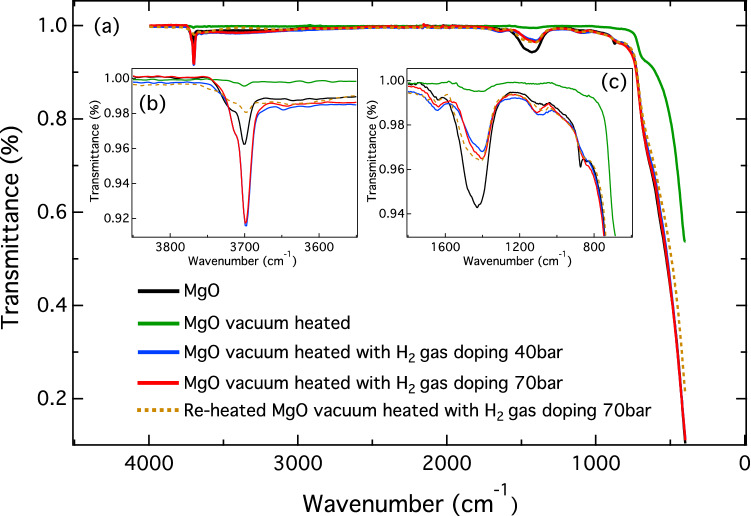
(**a**) FTIR spectra of MgO (solid black line), vacuum heated MgO (solid green line), MgO vacuum heated with H_2_ gas at 40 bar (solid blue line), MgO vacuum heated with H_2_ gas at 70 bar (solid red line), and reheated vacuum heated MgO with H_2_ gas at 70 bar (yellow dashed line). (**b**,**c**) Magnified FTIR peaks at approximately 3700 cm^−1^ for–OH stretching vibrations (**b**) and peaks at approximately 1500 cm^−1^ for physisorbed water bending modes (**c**).

To confirm the presence of hydrogen in MgO, we measured FTIR spectra for the MgO commercial powder, vacuum heated MgO, MgO vacuum heated with H_2_ gas at 40 bar and 70 bar, and the reheated vacuum heated MgO with H_2_ gas at 70 bar. The FTIR spectra of all MgO samples recorded over the wavenumber range 0–4500 cm^−1^ are shown in Fig. 8a, which shows two strong absorption peaks at 3700 cm^−1^ and 1500 cm^−1^. The absorption peak at 3700 cm^−1^ corresponds to a –OH stretching vibration in MgO^46^, whereas the absorption peak at 1500 cm^−1^ is related to bending vibrational modes of physisorbed water^47^. These observations may have resulted from reactions between all MgO samples and humidity in the ambient environment during sample preparation for FTIR measurements. The presence of the –OH vibration is consistent with our calculation and the VSM results, as discussed earlier. The MgO commercial powder (solid black line) showed an absorption peak at 3700 cm^−1^, which was higher than that of the vacuum-heated MgO sample (solid green line), as shown in Fig. 8b. This indicated that the MgO commercial powder had already absorbed hydrogen, which might have come from humidity in the ambient environment during sample preparation, as indicated by a very intense physisorbed water peak at 1500 cm^−1^. After heat treatment, we found that the vacuum heated MgO sample showed the weakest absorption peak at 1500 cm^−1^ (see Fig. 8c), implying that the physisorbed water was removed and the structure was changed due to intrinsic defects such as V_Mg_. After doping the vacuum heated MgO sample with H_2_ gas, we observed that the absorption peaks at 3700 cm^−1^ for MgO vacuum heated with H_2_ gas at 40 bar and 70 bar (blue and red solid lines) were substantially increased, which was evidence of hydrogen absorption by the samples (see Fig. 8b). Hydrogen absorption was further confirmed by reheating the sample in a vacuum at 550 °C. The reheated MgO sample doped with H_2_ gas at 70 bar (yellow dashed line) showed a decrease in the absorption peak at 3700 cm^−1^ (see Fig. 8b), indicating that adsorbed hydrogen was eliminated by the heating process.

## Conclusion

We used first-principles DFT calculations to investigate formation and the electronic and magnetic properties of MgO samples containing intrinsic vacancy defects and added hydrogen impurity atoms. We found that magnetic moments cannot exist in a perfect MgO crystal or the hydroxide Mg(OH)_2_. Unpaired electrons dominate the formation of magnetic moments in MgO from V_Mg_, which was confirmed by the formation energy and density of states. Moreover, a hydrogen impurity can be formed as an interstitial (H_i_) in MgO and is likely to form a ${\mathrm{H}}_{\mathrm{i}}-{\mathrm{V}}_{\mathrm{Mg}}$ complex. The magnetic moment of ferromagnetic V_Mg_-MgO is suppressed by hydrogen in the ${\mathrm{H}}_{\mathrm{i}}-{\mathrm{V}}_{\mathrm{Mg}}$ form and donated to unpaired electronic states. Therefore, H causes the reduced and degraded magnetic properties in ferromagnetic MgO.

The XRD result showed that new small peaks seen at approximately 35°, 50°, and 60° after heat treatment and hydrogen doping correspond to vacancy defects and hydrogen impurities in the MgO structure. The VSM result confirms that the vacuum-heated MgO sample exhibits the highest ferromagnetic magnetization induced by V_Mg_. The ferromagnetism of V_Mg_-MgO can be suppressed by hydrogen doping, as ferromagnetic magnetism is decreased by increased doping with H_2_ gas, which results from the formation of ${\mathrm{H}}_{\mathrm{i}}-{\mathrm{V}}_{\mathrm{Mg}}$. The presence of doped hydrogen in MgO was confirmed by FTIR measurements. The FTIR result also shows that the hydrogen dopant in MgO can be eliminated by heating. Our findings demonstrate that the ferromagnetic and diamagnetic properties of MgO can be controlled by heat treatment and hydrogen doping, which is useful for applications in magnetic sensing and switching.

## Data Availability

Experimental and calculated data are available for access at https://1drv.ms/u/s!AvS-bfeefGHCjR36okvsPenSIgim?e=Xk4f26.
